# Idiopathic harlequin syndrome: a case report and literature review

**DOI:** 10.11604/pamj.2019.33.141.18102

**Published:** 2019-06-25

**Authors:** Khadija Elboukhari, Hanane Baybay, Sara Elloudi, Zakia Douhi, Fatima Zahra Mernissi

**Affiliations:** 1 Departement of Dermatology, University Hospital of Fez, Morocco

**Keywords:** Harlequin syndrome, sympathetic system, anidrosis

## Abstract

Harlequin's syndrome is a rare dysautonomic syndrome of the face characterized by sweating with flush of one side and anhidrosis of the contralateral side. Mostly idiopathic although several secondary cases have been reported in the literature, the purpose of the treatment is mainly aesthetic and functional. We report the case of a patient having harlequin syndrome in its idiopathic form with a literature review.

## Introduction

Harlequin syndrome, first described in 1988 by Lance [1], is a dysautonomic syndrome of the face due to a unilateral dysfunction of the sympathetic system. It is more common in women, and most often benign and idiopathic. An organic cause may exist in one out of six patients, when associated with dysautonomic syndromes such as Claude Bernard Horner, it is called a harlequin sign [2]. The key symptom for consultation is the localized and often idiopathic anidrosis of the hemiface. In our article, we will present the case of idiopathic Harlequin syndrome with a review of the literature.

## Patient and observation

**Figure 1:**
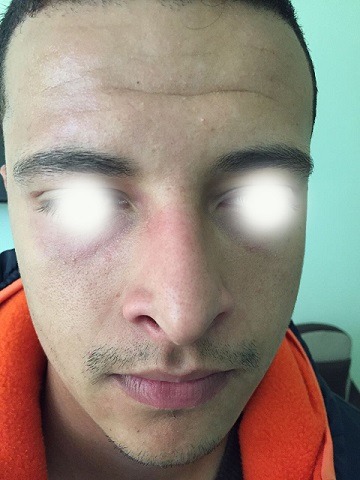
Right hemifacial erythema with sweat

**Figure 2:**
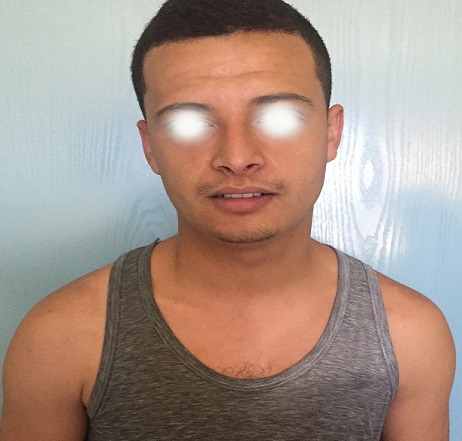
No sweat on the left side of the face, neck and superior zone of the trunk

Our patient is 21 years old, having as antecedents an essential epilepsy, autoregressive at the age of 18 years. Elsewhere, the patient has no history of diabetes or neuropathy, no previous cervicothoracic surgery or arterial catheterization, no history of trauma. He consulted for hypersudation and asymmetrical erythema of the face, lateralized on the right side. This disorder was noted since childhood, during sports efforts and more frequently in the hot season, otherwise the patient does not report other signs including ocular or neurological. The dermatological examination did not show abnormalities, no asymmetry of the face. The neurological examination, including the higher functions were normal, the examination of the cranial pairs especially the facial nerve was unscathed. In order to objectivate the disorder communicated by the patient, a stress test was carried out: after 20-minute race, an erythema appears of the right side of his face, neck and limb was demonstrated (Figure 1), more accentuated in suborbital, where it is finely telangiectatic, with hypersudation contrasting with the absence of erythema and sweat on the contralateral side (Figure 2). The diagnosis of the harlequin syndrome was then retained, and a cervicothoracic Magnetic resonance imaging (MRI) was performed not objectifying any abnormalities.

## Discussion

The sweat and facial vasomotor innervation are provided by sympathetic fibers whose origin is hypothalamus that will synapses with the fibers of the lateral horn of the spinal cord at the T2-T3 roots [3, 4]. The harlequin syndrome is then the expression of an abnormality of the sympathetic nervous system, originating from the T2-T3 roots or their nerve fibers, the site of the lesions may be at the level of sympathetic and parasympathetic nerve fibers of the stellate ganglion. It is a unilateral blocking of sympathetic innervation with absence of cutaneous vasodilatation and sweat secretion in response to a thermal, emotional or other stimulus [5]. This causes compensatory contralateral sympathetic hyperstimulation, explaining hypersudation with a flush. This syndrome is triggered most often after exercise and following exposure to heat, as reported by our patient. Clinically, it corresponds to a unilateral facial erythrema with flush and hypersudation [6] giving the appearance of a harlequin face reminiscent of the black red mask of Arlequin, well known in European folklore [7].

The absence of erythema or sweats on one side of the hemiface should make us look for an anomaly on this side [8], several etiologies have been reported, at the top of the list tumors of the cervicobrachial plexus [9], like neuroblastoma. A reported case of harlequin syndrome has been described in an adult associated with hypochromia of the iris, with the underlying anomaly being C1L1 nerve compression [4]. The rest of the etiologies are dominated by diabetic neuropathies, Guillain Barré syndrome, Bradbury Egleston syndrome, toxic goiter [9] cervicobrachial plexus pathology [9], medullary infarct, carotid dissection [3] cervical lymphatic malformation. However, this syndrome can rarely complicate cervico-spinal surgery [10].

As it can remain most often idiopathic and that was the case of our patient. Indeed, on a review of literature, published by Guillotton *et al.*, 59 out of 108 cases were idiopathic [11]. The spontaneous regression of this primitive syndrome has not been reported and studies are evolving in order to better understand its physiopathology [4]. Therapeutically and in order to preserve the aesthetic concern, periodic injections of botulinum toxin on the healthy side can be proposed, this treatment which remains the least invasive compared to the ipsilateral sympathectomy or the repetitive blocking of the stellate ganglion [12, 13] our patient has opted for this therapeutic method once reassured of the benign nature of this disorder.

## Conclusion

The harlequin syndrome is a dysautonomic phenomenon; most often idiopathic especially if the occurrence is early, the presence of neurological associated or cardiovascular signs must make look for an underlying cause especially in the adult. A knowledge of this syndrome by the dermatologist allows a precise diagnosis and to reassure the patient. In this perspective, a regular follow-up on our patient, and a botulinum toxin injection was considered per six months, so we will judge the effectiveness and cost-effectiveness of this alternative treatment that will make the object of another job.

## Competing interests

The authors declare no competing interests.
